# Acellular Dermal Matrix Prevents Esophageal Stricture After Full Circumferential Endoscopic Submucosal Dissection in a Porcine Model

**DOI:** 10.3389/fbioe.2022.884502

**Published:** 2022-05-02

**Authors:** Baozhen Zhang, Yue Zhang, Yidan Wang, Fan Yang, Shiyun Sheng, Zhe Wang, Xiaoying Chang, Jianyu Wei, Jintao Guo, Siyu Sun

**Affiliations:** ^1^ Department of Gastroenterology, Shengjing Hospital of China Medical University, Shenyang, China; ^2^ Department of Pathology, Shengjing Hospital of China Medical University, Shenyang, China; ^3^ Medical Transformation Department, Micro-Tech (Nanjing) Co. Ltd., Nanjing, China

**Keywords:** acellular dermal matrix, fully covered self-expanding metal stents, endoscopic submucosal dissection, esophageal stricture, regenerative medicine

## Abstract

Esophageal stricture is a common complication after endoscopic submucosal dissection (ESD), especially in full circumferential ESD. This study investigated fully covered self-expanding metal stent (FCSEMS) placement with an acellular dermal matrix (ADM) for preventing post-ESD esophageal stricture. Twelve Bama minipigs were randomly divided into two groups, which underwent full circumferential ESD in the distal esophagus. In group A, an FCSEMS with ADM was placed at the mucosal defect, whereas group B underwent standard FCSEMS placement. The stent was removed during gastroscopy 2 weeks after the ESD procedure. At the fourth week, gastroscopy was repeated to evaluate local healing and stenosis. The animals were sacrificed, esophageal specimens were obtained for macroscopic and histological evaluation, and serum C-reactive protein (CRP) levels were quantified. Four weeks post ESD**,** dysphagia occurrence was lesser in group A than in group B. Group A demonstrated lesser esophageal stricture on macroscopic evaluation (21.02 ± 16.65% vs. 57.41 ± 8.48%, *p* = 0.001) in the form of enhanced re-epithelization (99.13 ± 0.98% vs. 96.63 ± 1.64%, *p* = 0.009), diminished submucosal fibrosis (1117.53 ± 188.83 um vs. 1834.69 ± 421.99 um, *p* = 0.003), and attenuated inflammatory infiltration (121.00 ± 30.66 vs. 188.17 ± 64.92, *p* = 0.045). The increase in the serum CRP level was lower in group A than in group B at 4 weeks post-ESD. FCSEMS combined with ADM can enhance re-epithelization in the process of wound healing and significantly reduce the degree of esophageal stenosis after circumferential ESD. This study provided important preclinical findings for subsequent clinical trials.

## Introduction

Esophageal cancer is a common malignant tumor of the digestive tract (Qiu et al., 2021; Sung et al., 2021). With advancements in endoscopic technologies, more esophageal diseases can be diagnosed and treated through endoscopy (Rabinowitz et al., 2020a; Rabinowitz et al., 2020b; Tang et al., 2020; Hoibian et al., 2021). Endoscopic submucosal dissection (ESD) has become the preferred therapy for early intramucosal esophageal cancers (Probst et al., 2015; Qi et al., 2018), especially because the R0 resection rates are higher. However, the cardinal long-term complication of ESD is esophageal stricture (Mizuta et al., 2009; Isomoto et al., 2013). In particular, an esophageal mucosal defect occupying more than three-quarters of the lesion circumference is an independent risk factor for esophageal stricture after ESD (Tang et al., 2021; Zhang et al., 2021). It is worth noting that almost all patients who undergo full circumferential ESD eventually develop esophageal stenosis (Yu et al., 2019). Currently, the rate of esophageal stricture prevention is unsatisfactory (Bhatt and Mehta 2020; Zhang et al., 2020).

With tissue engineering and regenerative medicine approaches, including the use of biologic scaffolds or cell-based therapies, researchers are exploring treatments that can reconstruct structurally and functionally normal tissues. One of such novel treatments comprises the use of an acellular dermal matrix (ADM)—a type of bioresorbable membrane—that showed effectiveness in previous animal experiments for providing local coverage of the mucosal defect in preventing esophageal stenosis after hemi-circumferential ESD (Han et al., 2017). Nevertheless, stenosis after full circumferential ESD requires viable solutions, and the effectiveness of ADM in circumferential ESD could not be verified until now. The application of an ADM in full circumferential ESD is challenging, because ADM fixation in a circumferential excision wound is difficult.

Therefore, we developed a new type of fully-covered self-expanding metal stents (FCSEMS) with an attached ADM and aimed to evaluate its feasibility of application and efficacy for esophageal stenosis prevention after full circumferential ESD in a porcine model.

## Materials and Methods

### Study Design

All animals were either assigned into group A (*n* = 6) or group B (*n* = 6) using a random digits table. Animals in both groups underwent full circumferential esophageal ESD. Immediately after the ESD procedure, endoscopic placement of FCSEMS with ADM was conducted in group A, whereas standard FCSEMS placement was performed in group B. Prior to the procedure, we conducted tests to validate that the formation of esophageal stenosis could be established post-ESD without any intervention.

### Animals

The experimental protocol was approved by the Ethics Review Committee of Shengjing Hospital of China Medical University (No. 2021PS030K). Male Bama mini pigs weighing between 12 and 15 kg received a 48-h half-liquid diet, and fasted for 24-h before the procedure. Thereafter, animals were obtained by the Laboratory Animal Science Department of Shengjing Hospital. Anesthesia was induced by an intravenous propofol injection; venous access was established through the marginal ear vein. Ventilation was maintained via endotracheal intubation. All procedures were performed with the animals under continuous electrocardiographic monitoring.

### Circumferential Endoscopic Submucosal Dissection

**FIGURE 1:**
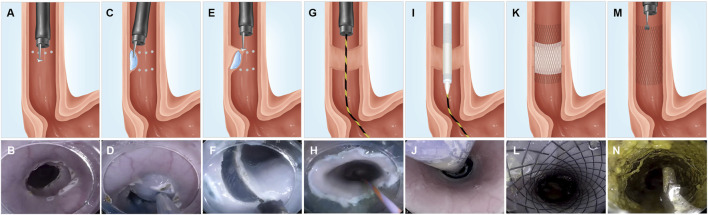
Representative illustrations and corresponding endoscopic views of the ESD procedure and FCSEMS with ADM placement and removal. **(A–B)** We marked the full circumference on both the cranial and caudal sides, at approximately 30-cm distance from the incisors. **(C–D)** Submucosal injection of methylene blue and saline between the full circumferential markings of the cranial and caudal sides of an esophageal lesion. **(E–F)** Submucosal dissection performed with a triangular knife. **(G–H)** Placement of a guidewire under endoscopic guidance after full circumferential ESD. **(I–J)** A stent conveyer is inserted along with the guidewire, and the FCSEMS with ADM was placed by driving the stent conveyer. **(K–L)** Outcome of FCSEMS with ADM placement. **(M–N)** Stent removal using forceps. FCSEMS, fully covered self-expanding metal stent; ADM, acellular dermal matrix.

The anesthetized mini pigs were placed in the left lateral position. All the procedures were performed using a gastroscope (EPK-I, Pentax, Tokyo, Japan) with a transparent cap. We marked the circumference of the lesion on both the cranial and caudal sides of the esophagus, at approximately 30 cm distance from the incisors. Following a submucosal injection of methylene blue and saline, submucosal dissection of 1-cm-length was performed on the local mucosa using a triangular knife (KD-640L, Olympus, Tokyo, Japan) (Figures 1A–F). Hot coagulation forceps (FD-410LR, Olympus, Tokyo, Japan) were used for ensuring hemostasis.

### Preparation of Fully Covered Self-Expanding Metal Stent With Acellular Dermal Matrix and Stent Placement

**FIGURE 2:**
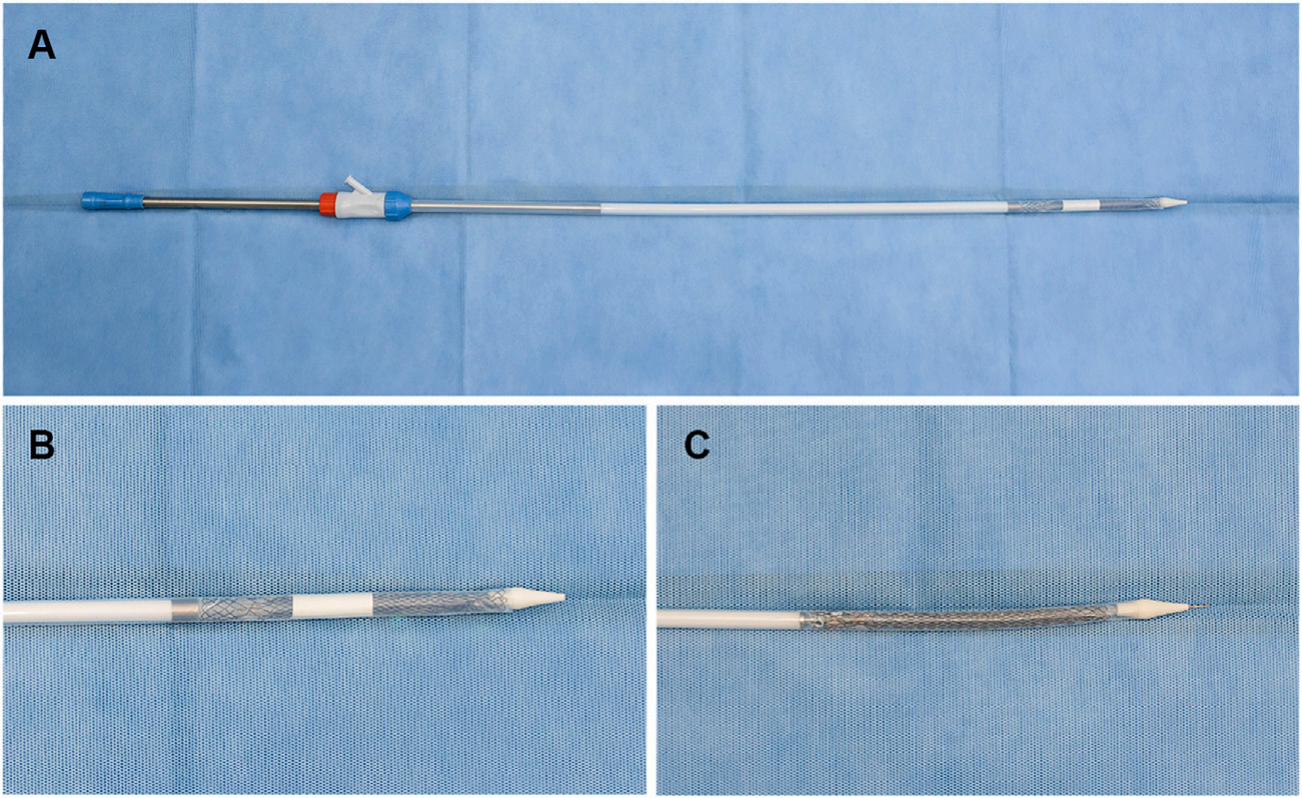
Representative images showing FCSEMS with ADM and standard FCSEMS. **(A,B)** Outcome of FCSEMS with ADM: FCSEMS is covered by the ADM in the middle and upper part of the stent and is inserted in the stent conveyer. **(C)** The appearance of FCSEMS. FCSEMS, fully covered self-expanding metal stent; ADM, acellular dermal matrix.

The ADM membrane (Yantai Zhenghai Bio-tech Co., Ltd., Yantai, China) has been derived from meshed bovine skin, which was sterilized and decellularized using proprietary bioengineering methods. We used the ADM membrane to cover the wound post-ESD using an esophageal FCSEMS (Micro-Tech [Nanjing] Co., Ltd., Nanjing, China) (Figures 2A,B). A standard FCSEMS device was also procured from Micro-Tech (Nanjing) Co., Ltd. (Figure 2C).

Immediately after full circumferential ESD, a guidewire was placed under the endoscope, the endoscope was pulled out, and a stent conveyer (Supplementary Figure S1) was introduced along with the guidewire for stent placement. The endoscope was reinserted to observe if the stent was positioned to cover the mucosal defect (Figures 1G–L) completely.

### Post-Endoscopic Submucosal Dissection Care and Stent Removal

Intravenous antibiotics (sulperazon, 0.75 g/d) were administered to all animals for 1 day and a proton pump inhibitor (esomeprazole, 40 mg/d) was administered for 3 days post-ESD. The minipigs were fasted for 24 h after the ESD, and liquid diet was resumed on the second day post-ESD. A soft diet was provided for a week, which was gradually transitioned to a solid diet. Endoscopy was performed 2 weeks post-ESD, and the stent was removed using forceps in both groups (Figures 1M,N). A repeat gastroscopy was performed at week 4 post-ESD, after which all the minipigs were sacrificed using an overdose of propofol, and the esophagus of each minipig was retrieved for further assessment.

### Post-Endoscopic Submucosal Dissection Evaluation and Follow-Up

#### General Condition

For each animal, experienced veterinarians assessed the weight change, daily food intake, and vomiting status on a regular basis. Dysphagia severity was estimated using the Mellow-Pinkas dysphagia score (Kim et al., 2015) (0 = no dysphagia; 1 = dysphagia to normal solids; 2 = dysphagia to soft solids; 3 = dysphagia to solids and liquids; and 4 = complete dysphagia, even to saliva).

#### Endoscopic Evaluation

Gastroscopic evaluation of the esophageal stricture was performed, wherein mini pigs with dysphagia that resisted routine endoscope insertion (EPK-I, Pentax, Tokyo, Japan) into the esophagus were considered to have an esophageal stricture.

#### Macroscopic Evaluation

The harvested esophagus was incised longitudinally and fixed on a foam board, after which a macroscopic specimen was evaluated. The degree of esophageal stricture (%) was calculated as follows:

$$\text{Extent}\;\text{of}\;\text{esophageal}\;\text{stricture}\left(\text{\%}\right)=\left[1-{\text{L}}_{\text{max}}/50\text{\%}\left({\text{L}}_{\text{a}}{+\text{L}}_{\text{b}}\right)\right]\text{×}100$$ (1)

where L_max_ represents the length of the narrowest regenerative mucosal short axis and L_a_ and L_b_ are the lengths of the normal mucosal short axes on the cranial and caudal sides of the esophageal specimen, respectively (Tang et al., 2018).

#### Histological Analysis

Following the macroscopic evaluation, the esophageal specimens were fixed in a 10% formalin buffered solution, embedded in paraffin, processed into sections, and then stained with hematoxylin-eosin and Masson’s trichrome. A senior pathologist, blinded to the treatment groups, analyzed all the slides. The border between the re-epithelialized tissue and the original mucosal epithelium was identified by the lack of muscularis mucosal tissue. The proportion of ESD ulceration covered with re-epithelialized tissue was evaluated using the below formula:

Ratio of re‐epithelization(%)=[RL/DL]×100% (2)

where RL and DL represent the lengths of the re-epithelialized tissue and the dissected mucosal long axis, respectively (Tang et al., 2018). The presence of fibrosis in the submucosal layer was indicated by the maximal thickness of the submucosal fibrotic tissue in the vertical direction. Semiquantitative analysis of the inflammatory cells was performed at 400 × magnification. Inflammatory cells between the epithelium and the muscular layer were counted in each of five randomly selected high-power fields (HPFs).

#### Pro-Inflammatory Marker Level Measurement

Blood samples were obtained at day 0, week 2, and week 4 post-ESD to measure the serum levels of the pro-inflammatory marker, CRP, in each specimen.

### Statistical Analysis

Statistical analyses were performed using SPSS 23.0 (IBM Corp, Armonk, NY, United States) and Microsoft Office 2019 (Microsoft Corporation, Redmond, United States). Figures were prepared using GraphPad Prism 8 (GraphPad Software, San Diego, United States). Data are presented as mean ± standard deviation or count (percentage). Comparisons between groups were performed using the Student’s *t*-test or Fisher’s exact test. A *p*-value < 0.05 was considered to be statistically significant.

## Results

### Procedure Results

**FIGURE 3:**
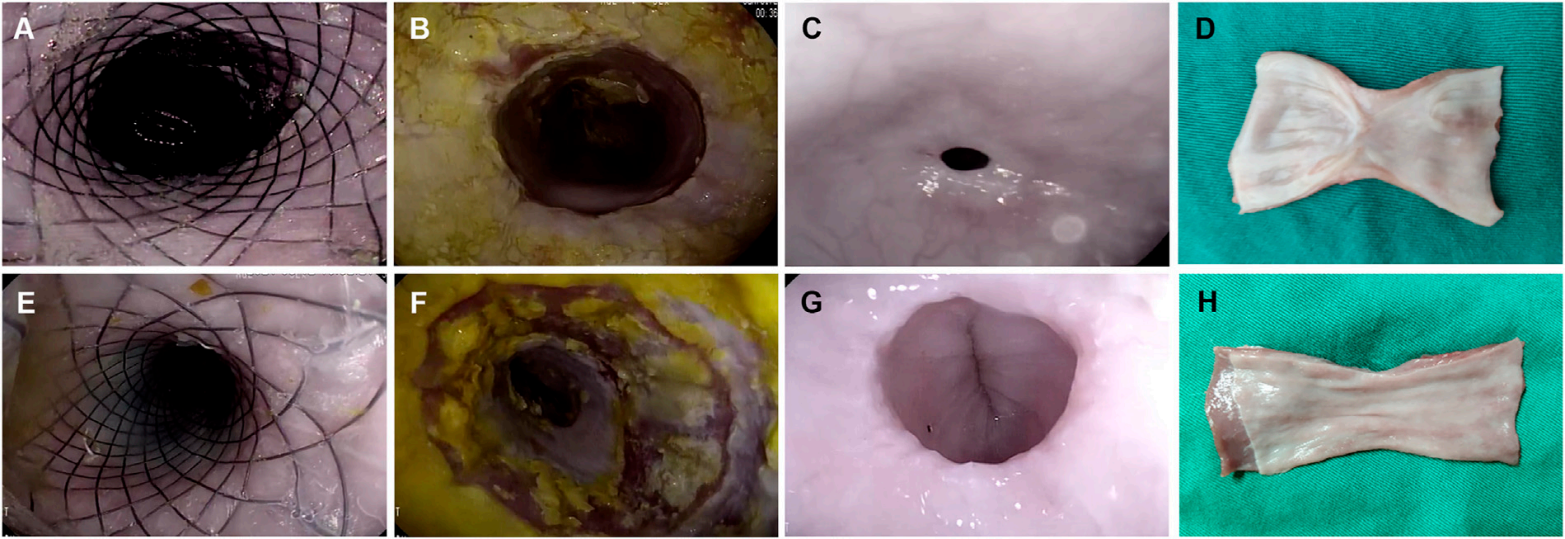
Comparison between the ADM-FCSEMS group **(A)** and FCSEMS group **(B)** by endoscopic and macroscopic evaluations. **(A,E)** Endoscopic appearance immediately after full circumferential endoscopic submucosal dissection (ESD) and stent placement in both groups. **(B,F)** Endoscopic appearance at week 2 post-ESD. No obvious esophageal strictures in groups A and B. **(C,G)** Endoscopic appearance at week 4 post-ESD in groups A and B. A greater esophageal diameter and better patency can be observed on endoscopy in group A than in group B. **(D,H)** Macroscopic views of the harvested esophageal specimen show better wound healing and lesser mucosal contraction in group A than in group B.

**FIGURE 4:**
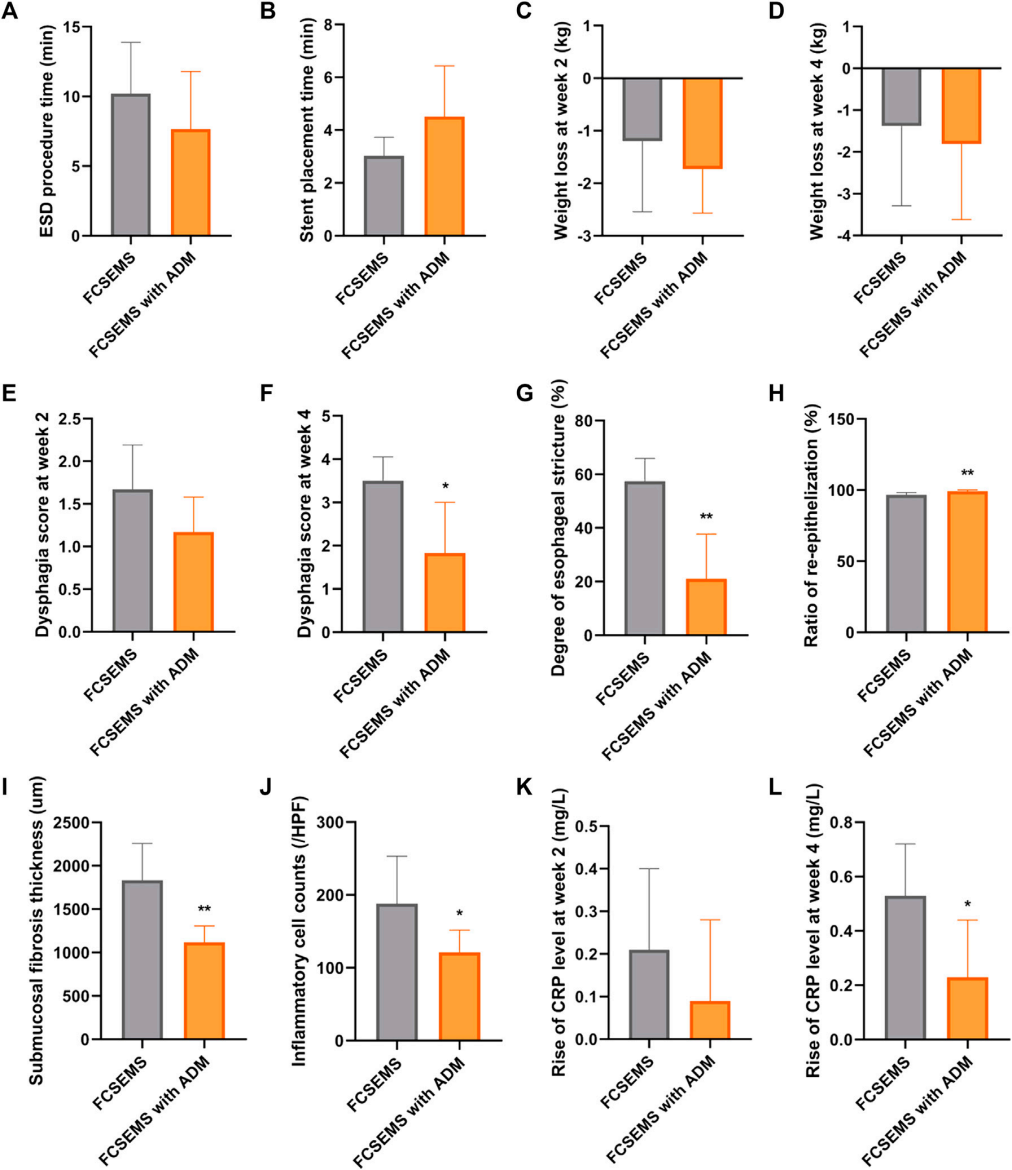
Data comparisons between the ADM-FCSEMS group **(A)** and FCSEMS group **(B)**. Comparisons of the **(A)** ESD procedure time, **(B)** stent placement time, **(C)** weight loss at week 2 post-ESD, **(D)** weight loss at week 4 post-ESD, **(E)** dysphagia score at week 2 post-ESD, **(F)** dysphagia score at week 4 post-ESD, **(G)** stricture degree in the macroscopic evaluation, **(H)** ratio of re-epithelization, **(I)** submucosal fibrosis thickness, **(J)** inflammatory cell counts, **(K)** rise of C-reactive protein (CRP) level at week 2 post-ESD, and **(L)** rise of CRP level at week 4 between both groups. Bars with an asterisk are indicating significant differences [*p* < 0.05 (∗), *p* < 0.01 (∗∗)].

In the study, 12 male Bama minipigs weighing 13.34 ± 1.44 kg were randomly assigned into group A (*n* = 6) or group B (*n* = 6). Table 1 presents the results of procedure. Full circumferential ESD was successfully performed in both groups (12/12, 100%) (Figures 3A,E). The mean length of the longitudinal esophageal mucosal defect in all minipigs was 2.58 ± 0.51 cm. All the animals in groups A and B survived postoperatively. There were no significant differences between the two groups in the lengths of mucosal defect, duration of ESD and stent placement (Table 1; Figures 4A,B).

**TABLE 1:** ESD procedure and stent placement data in both groups.

	Group A (*n* = 6)	Group B (*n* = 6)	*p*-Value
Mucosal defect length (cm)	2.67 ± 0.52	2.50 ± 0.55	0.599^a^
ESD procedure time (min)	7.66 ± 4.13	10.21 ± 3.67	0.284^a^
Stent placement time (min)	4.52 ± 1.91	3.03 ± 0.70	0.102^a^
En bloc resection	6 (100%)	5 (83.33%)	0.500^b^
Bleeding	0	1 (16.67%)	0.500^b^
Perforation	0	0	-

a Student’s *t*-test.

b Fisher’s exact test.

Group A minipigs underwent FCSEMS with ADM placement; Group B minipigs underwent FCSEMS placement.

ESD, endoscopic submucosal dissection.

### General Condition

As shown in Table 2 and Figures 4C, D, there was no significant difference in the weight loss post-ESD between groups A and B at week 2 and week 4. Although the food tolerability post-ESD was comparable between groups A and B at week 2, animals in group A had better food tolerability than those in group B at week 4 (Table 2; Figures 4E,F).

**TABLE 2:** Physical, endoscopic, histologic, and biological comparisons between both groups.

	Group A (*n* = 6)	Group B (*n* = 6)	*p*-Value
General condition			
Weight loss (kg, post-ESD wk 2)	−1.73 ± 0.84	−1.20 ± 1.34	0.435^a^
Weight loss (kg, post-ESD wk 4)	−1.81 ± 1.81	−1.38 ± 1.91	0.701^a^
Dysphagia score (post-ESD wk 2)	1.17 ± 0.41	1.67 ± 0.52	0.092^a^
Dysphagia score (post-ESD wk 4)	1.83 ± 1.17	3.50 ± 0.55	0.010^a^
Endoscopy evaluation			
No. of stricture cases (post-ESD wk 2)	0 (0%)	0 (0%)	-
No. of stricture cases (post-ESD wk 4)	1 (16.67%)	5 (83.33%)	0.039^b^
Macroscopic evaluation			
Stricture degree (%)	21.02 ± 16.65	57.41 ± 8.48	0.001^a^
Histological evaluation			
Ratio of re-epithelization (%)	99.13 ± 0.98	96.63 ± 1.64	0.009^a^
Submucosal fibrosis thickness (um)	1117.53 ± 188.83	1834.69 ± 421.99	0.003^a^
Inflammatory cell counts/ HPF	121.00 ± 30.66	188.17 ± 64.92	0.045^a^
CRP levels			
Rise of CRP level (mg/L, post-ESD wk 2)	0.09 ± 0.19	0.21 ± 0.19	0.283^a^
Rise of CRP level (mg/L, post-ESD wk 4)	0.23 ± 0.21	0.53 ± 0.19	0.027^a^

a Student’s *t*-test.

b Fisher’s exact test.

Group A minipigs underwent FCSEMS with ADM placement; Group B minipigs underwent FCSEMS placement.

ESD, endoscopic submucosal dissection; HPF, high-power field; CRP, C-reactive protein; No, number; FCSEMS, fully covered self-expanding metal stent; ADM, acellular dermal matrix.

### Endoscopic Evaluation

From the gastroscopy evaluations, the frequency of esophageal stricture in group A was lower than that in group B at week 4 (16.67 vs. 83.33%, *p* = 0.039) (Table 2; Figures 3C,G); however, there was no stricture occurrence in both groups at week 2 (0 vs. 0%) (Table 2; Figure 3B,F).

### Macroscopic Evaluation

On macroscopic evaluation of esophageal specimens at week 4, the mean reduction in the luminal circumference of the esophagus for groups A and B was 21.02% (range: 1.75–49.02%) and 57.41% (range: 48.15–73.13%), respectively (Table 2; Figures 3D, H and Figure 4G). This outcome was consistent with the dysphagia scores and endoscopic examinations in both groups at week 4 post-ESD.

### Histological Evaluation

**FIGURE 5:**
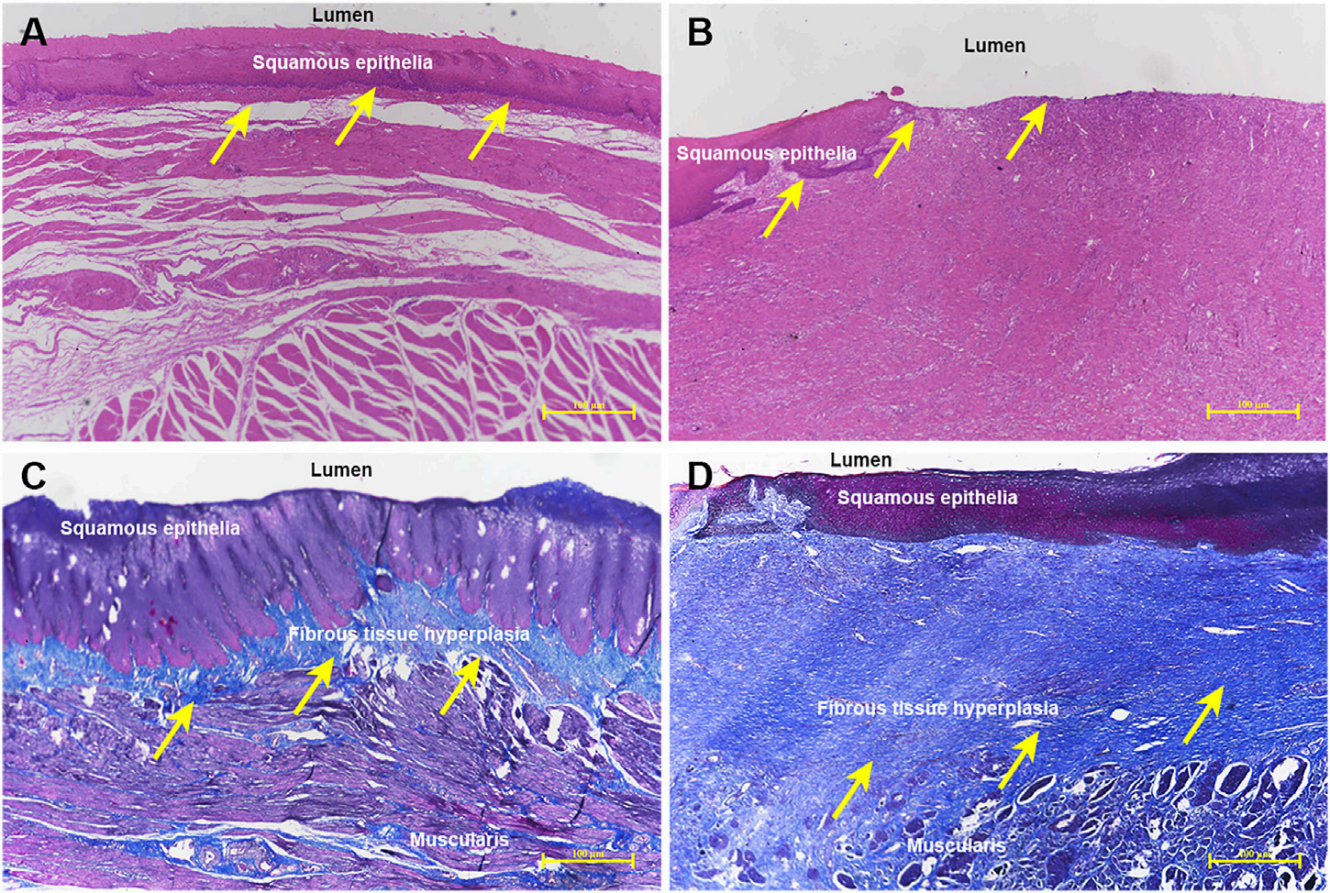
Histological comparison of the ADM-FCSEMS group **(A)** and FCSEMS group **(B)**. Hematoxylin-eosin staining reveals **(A)** an ESD wound covered by abundant continuous and integrated neoepithelium in group A (yellow arrows, ×40) and **(B)** irregular and unattached re-epithelium in the process of wound healing in group B (yellow arrows, ×40). Masson’s trichrome staining demonstrates **(C)** less submucosal fibrosis in group A (yellow arrows, 40×) and **(D)** obvious submucosal fibrosis formation and structural disorder in group B (yellow arrows, ×40).

All histological results are reported in Table 2 and Figure 4H–J, and Figure 5A–D. Group A had a higher re-epithelialization rate than that of group B (99.13 ± 0.98% vs. 96.63 ± 1.64%, *p* = 0.009). The maximum thickness of the fibrosed submucosal layer was significantly smaller in group A than in group B (1117.53 ± 188.83 um vs. 1834.69 ± 421.99 um, *p* = 0.003). There were fewer inflammatory cells per HPF at the remodeled ESD site in group A than in group B (121.00 ± 30.66 vs. 188.17 ± 64.92, *p* = 0.045).

### Serum C-Reactive Protein Levels

There was no significant difference in the increase in CRP levels between both groups at week 2 post-ESD (0.09 ± 0.19 mg/L vs. 0.21 ± 0.19 mg/L, *p* = 0.283) (Table 2; Figure 4K). At week 4 post-ESD, the increase in CRP levels in group A was significantly smaller than that in group B (0.23 ± 0.21 mg/L vs. 0.53 ± 0.19 mg/L, *p* = 0.027) (Table 2; Figure 4L).

## Discussion

Prior to conducting our formal experiment, we first demonstrated that in the absence of intervention, the mucosal defect formed by 1-cm circumferential esophageal ESD could lead to esophageal stenosis in a porcine model. All the mini pigs developed esophageal stricture without the ability to tolerate oral intake, thus requiring euthanasia between postoperative weeks 2 and 3. On endoscopy, we found pinhole strictures and an obvious absence of epithelial tissue at the ESD site (Supplementary Figures S2A–S2B). Moreover, the pathological assessment of the esophageal specimens revealed a lack of continuous epithelium accompanied by significant submucosal fibrosis (Supplementary Figures S2C–S2D), suggesting that the establishment of full-circumference ESD by the above method can lead to esophageal stenosis without any intervention.

The allocation of the 12 minipigs was random between both the groups. There were no significant differences in the dysphagia score and stenosis 2 weeks after ESD between the two groups. At week 4 post-ESD, the animals in group A had lesser dysphagia score than those in group B. The reduction in the luminal circumference of the esophagus in group A was lower than that in group B, which was in line with the endoscopic results. The macroscopic evaluation indicated that ADM could decrease esophageal mucosal contracture by approximately 40%.

In group A, the pathological analyses revealed a well-regulated neo-epithelium, lesser submucosal layer fibrosis and inflammatory infiltration, and greater re-epithelization rate than those in group B. These findings could be explained by the efficacy of the ADM patch. One possible explanation is that the matrix can trap and bind the recipient’s own cells and growth factors, which favors the growth of esophageal epithelial cells and vascularization. Further, this can help achieve rapid epithelialization in the mucosal defect (Rennert et al., 2013; Xu et al., 2021). Simultaneously, in the wound area, the matrix can inhibit the fibrotic formation and infiltration towards the deeper muscular layer, which can effectively prevent muscularis propria injury and is important for tissue repair and reconstruction.

Since the mean CRP rise in group A was lower than that in group B at week 4 after ESD, the covering of mucosal defects by ADM may have an anti-inflammatory effect in circulation. The degree of CRP level increase is positively correlated with the degree of infection. Hence, from our comprehensive analysis of the local inflammatory cell counts and general elevation in CRP levels, the ADM patch was deemed capable of attenuating the inflammatory infiltration in the local defect and inflammatory reaction in the circulation. ADM has several potential advantages relating to its source tissue and manufacturing process, which may have contributed to the limited inflammatory response observed in this study. ADM is manufactured with all lipids, fats, carbohydrates, and non-collagen removed, thus retaining only collagen. This can minimize the inflammatory reactions and host immune response, resulting in less tissue damage due to inflammatory cell aggregation (Cornwell et al., 2009; Lantis et al., 2021).

Extrapolating from the evidence in our study, we speculate that ADM limits the formation of scar tissue and reduces esophageal stricture rate, possibly via diminished submucosal fibrosis, enhanced re-epithelization, and attenuated inflammatory cell/mediator infiltration.

This is the first study to evaluate the efficacy and safety of FCSEMS with ADM for the prevention of esophageal stricture. We conducted a robust post-endoscopic stricture validation with full circumferential wounds in a porcine model, while most studies have utilized a semi-circumferential ESD or smaller wounds. Moreover, ADM and FCSEMS were stably consolidated, and ADM could be placed at the wound site with the stent intraoperatively, where no metal clip or fibrin glue was required to secure the ADM graft attachment, which greatly decreased the complexity of ADM application. Simultaneously, we carried out a longer observation period (4 weeks) for acquiring more data.

While ESD is a common treatment option for early esophageal cancer, post-procedure stenosis has remained as its primary limitation. Moreover, the mechanisms underlying the development of an esophageal stricture post ESD remain to be comprehensively elucidated. Typically, the repair of post-ESD esophageal mucosal defect involves three phases: inflammatory response, epithelial proliferation, and extracellular matrix remodeling (Broughton et al., 2006). Honda et al. (2010) performed an animal study, from which they speculated that fibrosis formation occurs in the submucosal and muscularis propria during the mucosal defect healing process, which results in the decrease of the esophageal wall elasticity and movement. Consequently, esophageal stenosis is formed following this cascade. Thus, an effective prophylactic maneuver for an esophageal stricture is aimed at maintaining or re-establishing wound equilibrium and allowing the satisfactory healing process to occur by inhibiting the inflammatory response, promoting epithelial regeneration, and preventing muscularis propria injury.

Currently, tissue engineering and regenerative medicine approaches are targeted toward the reconstruction of structurally and functionally normal tissues, including tissue shielding methods with polyglycolic acid (PGA) sheets and fibrin glue (Sakaguchi et al., 2016), or autologous tissue transplantation (Chai et al., 2019). PGA sheet, a biodegradable suture material, can provide plentiful cytoskeletons that help in drug loading and cell crawling during the repair process, thereby leading to a decreased risk of scar germination and stricture formation (Chai et al., 2018). Nevertheless, these approaches require laborious methods to prepare shielding materials, and are associated with an unstable and complex technology.

ADM is a soft tissue graft created by the decellularization of tissue, which leaves the extracellular matrix in its original undamaged state while removing all materials which could potentially trigger a host immune response (Cornwell et al., 2009). In the current study, the ADM was available from bovine dermis, containing predominantly type I collagen. Additionally, it is produced under aseptic conditions, ensuring that the allograft is sterile. The ADM has been used successfully for wound healing and tissue repair, as well as tissue reconstruction (Jansen et al., 2013; Kirsner et al., 2015). However, few studies have focused on the repair of the gastrointestinal mucosal layer, particularly that of the esophagus. In our preliminary study (Han et al., 2017), we demonstrated the safety and efficacy of placing ADM with metal clips in a porcine model to prevent esophageal stricture formation after a semi-circumferential ESD; however, it was difficult to fix the ADM patch with metal clips on the circumferential wound.

Maintaining the organ morphology is of great significance for organ regeneration *in vivo*. To facilitate wound healing, the esophagus must be maintained in a tubular form for a certain period. Thus, the current study emphasizes how the ADM patch can be grafted on full circumferential wounds stably and easily, and how the esophageal tubular structure can be maintained. Therefore, we developed a novel stent, the ADM-FCSEMS, which provides the advantage of extraordinary water solubility of the ADM, which allows the adherence to moist wound surfaces with great rapidity. Therefore, no clip or fibrin glue is needed to secure sheet attachment. Additionally, ADM increases friction and adhesion between the stent and wound against stent migration, and the FCSEMS provides a radial force, which fixes the ADM in a stable manner and maintains the esophagus in a tubular form with minimal deformities.

The study was designed and conducted with careful attention to every process and material used; however, there were still several limitations. First, cases of mucosal defects formed after circumferential ESD with 2–3 cm lengths are uncommon in actual clinical settings. Considering the limited esophageal length in miniature pigs and our successful validation of stricture formation, we decided to resect 1-cm-long mucosa in the study. A mucosal defect of approximately 2-cm was formed due to esophageal ductility. Second, the consideration of weight variation as an evaluation outcome was imprecise, and despite development of obvious dysphagia at week 4 in Group B, weight loss in both groups was statistically insignificant. In addition, there was still one case of stenosis in group A at week 4, which may be related to the lack of new epithelium with sufficient thickness. Hence, our following study would focus on whether 2 weeks of stenting is appropriate and whether a longer period of stenting is needed.

## Conclusion

In conclusion, FCSEMS with ADM effectively prevented post-ESD esophageal stenosis possibly by the following mechanisms: diminished submucosal fibrosis, enhanced re-epithelization, and attenuated inflammatory cell/mediator infiltration at the microscopic level. We estimate that our results could provide important preclinical experience for subsequent clinical trials. Planning is underway for a first-in-human study to assess the feasibility of ADM-FCSEMS placement for the prevention of post-ESD esophageal stricture in patients with early esophageal cancer.

## Data Availability

The raw data supporting the conclusions of this article will be made available by the authors, without undue reservation.
