# Supplementary material for: Acellular Dermal Matrix Prevents Esophageal Stricture After Full Circumferential Endoscopic Submucosal Dissection in a Porcine Model
Source: Front Bioeng Biotechnol. 2022 May 2;10:884502. doi: 10.3389/fbioe.2022.884502 (PMC9108161; doi:10.3389/fbioe.2022.884502)
Supplement: Supplementary file 1 [file DataSheet1.PDF]

SUPPLEMENTARY MATERIAL

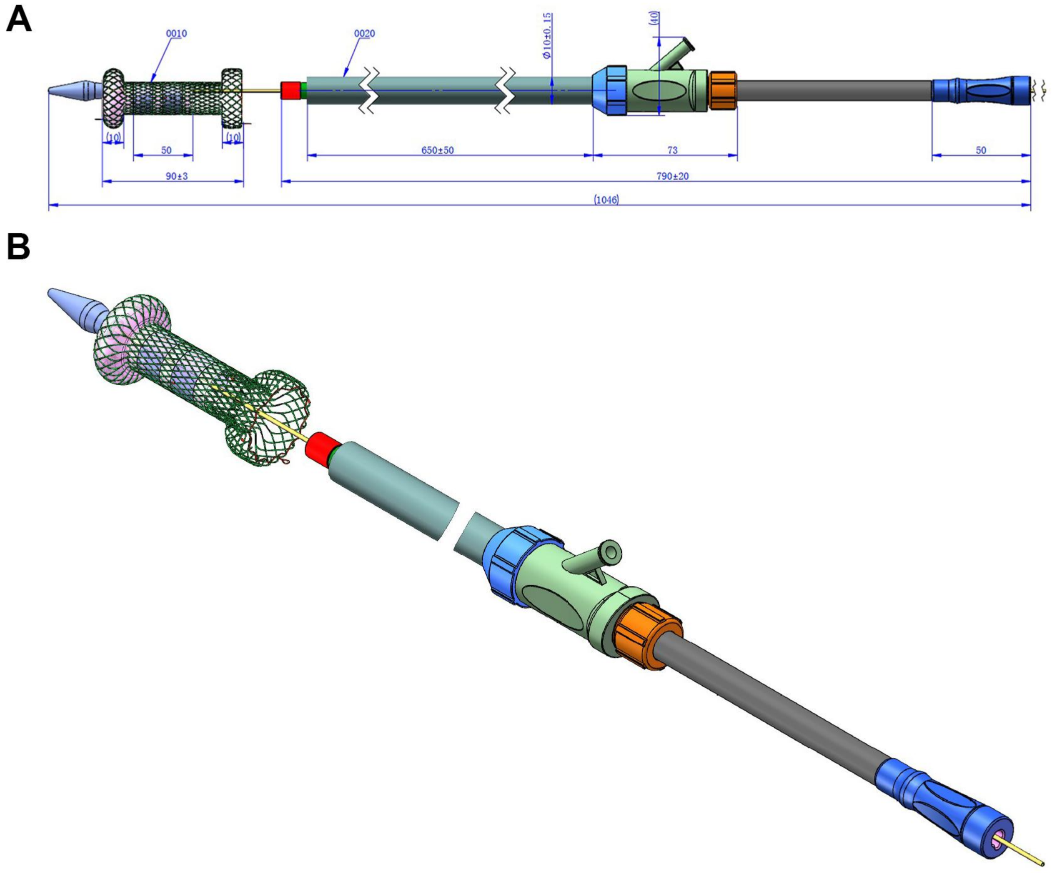

Figure S1. Schematic representation of the stent conveyer. (A) Dimensions and lengths of each part of the stent conveyer (unit of length: mm). (B) Structral illustration of stent conveyer.

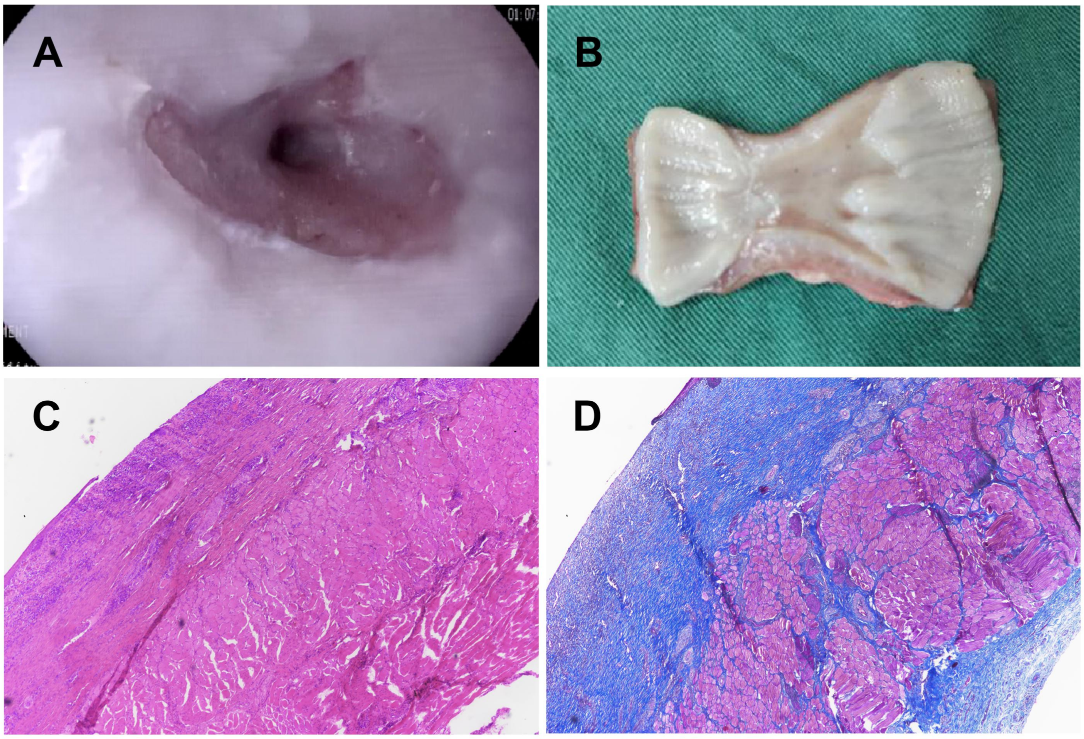

Figure S2. Validation tests that esophageal stricture formation could be established post-ESD without any intervention. (A) Endoscopic view 2 weeks after circumferential ESD. Esophageal pinhole strictures and an obvious absence of epithelial tissue were observed in three mini pigs. (B) Macroscopic view of the esophageal specimens demonstrates severe absence of esophageal epithelium. (C) Hematoxylin-eosin staining of esophageal specimens reveals epithelial disruption and lack of re-epithelium (40×). (D) Masson's trichrome staining shows diffuse fibrotic infiltration in the submucosal layer (40×).
